# Uncommon but Not Elusive: A Case of Syringocystadenoma Papilliferum in the External Auditory Canal

**DOI:** 10.7759/cureus.49685

**Published:** 2023-11-29

**Authors:** Tabitha Shirani, Suhana Rahim, Nur Eliana Ahmad Tarmizi, Avatar Singh Mohan Singh, Chua Hui Heng

**Affiliations:** 1 Department of Otorhinolaryngology-Head and Neck Surgery, Taiping Hospital, Taiping, MYS; 2 Department of Pathology, Taiping Hospital, Taiping, MYS

**Keywords:** ceruminous gland tumors, head and neck pathologies, head and neck neoplasms, external auditory ear canal, syringocystadenoma papilliferum

## Abstract

Ceruminous glands are modified apocrine sweat glands that are situated in the cartilaginous segment of the external auditory canal. Ceruminous gland tumors only account for about 5% to 5.7% of all external ear tumors. Syringocystadenoma papilliferum (SCAP) is a slow-growing and benign neoplasm that originates from the apocrine or eccrine sweat glands. It is also the rarest of all ceruminous gland neoplasms and only accounts for 2% of these cases. This is the 19^th^ reported case of SCAP in the external auditory canal in the English literature. The definite diagnosis of SCAP is confirmed histologically due to varied differentials from its clinical appearance. The mainstay treatment of SCAP is surgical excision of the tumor, in our case, we obtained an incisional biopsy to aid in the diagnosis and then proceeded with an excisional biopsy. The patient has no signs of tumor recurrence post-operatively. A detailed review of the clinical, radiographic, and histomorphological characteristics of SCAP is discussed.

## Introduction

Ceruminous glands are modified apocrine sweat glands found in the dermis of the cartilaginous section of the external auditory canal (EAC). Tumors of these glands are rare and represent only 5%-5.7% of all external ear tumors [1] while some authors have suggested an even lower frequency of these tumors [2]. Haugh was the first to report a neoplasm of the EAC ceruminous gland and coined the word ceruminoma in 1894 [3]. The World Health Organization officially classifies ceruminous gland neoplasms as benign ceruminous adenoma, ceruminous pleomorphic adenoma, and syringocystadenoma papilliferum (SCAP), as well as malignant adenocarcinoma, adenoid cystic carcinoma, and mucoepidermoid carcinoma [4]. Searches in PubMed, Google Scholar, and Science Direct were performed, there are less than 150 case reports of EAC-related ceruminous gland tumors in the literature [1] and only 18 cases of SCAP in the EAC have been presented; 18th case as reported by Jakovljevic et al. [3]. SCAP is a slow-growing, benign hamartomatous tumor that develops from the apocrine or eccrine sweat glands. It is mostly found in the scalp and face; involvement of the ear is rare [5]. SCAP is considered to be the rarest of the ceruminous gland neoplasms, accounting for only 2% of these tumors [6]. It was initially described in the dermatological community in the 20th century as a “naevus syringoadenomatous papilliferus” [7]. Neoplasms arising from the ceruminous glands of the EAC have provided a diagnostic challenge due to their nonspecific clinical and radiological features therefore definite diagnoses of SCAP primarily rely on histopathological examinations. In this report, we present a case of SCAP arising from the EAC with a review of its clinical, radiological, and histomorphological characteristics.

## Case presentation

A 70-year-old Malay male with no comorbidities presented with a right-sided worsening hearing loss due to a mass in the right ear. The painless mass had progressively grown in the past two years to obliterate the EAC in its entirety and extend laterally. It was also associated with intermittent hemoserous discharge; however, the patient did not have any tinnitus, vertigo, facial asymmetry, or any similar swelling elsewhere in the head and neck region. He denies any history of trauma, recurrent ear infections, and ear surgeries. The right EAC mass was polypoidal, friable, and bled upon contact. The mass was occupying the whole EAC till the meatal opening. No neck nodes were palpable on the ipsilateral cervical region. All cranial nerves were grossly intact. General and otoscopy examination of the left ear was unremarkable. Pure tone audiometry showed a profound mixed hearing loss on the right side with mild sensorineural hearing loss on the left side. A tissue biopsy of the mass was reported as SCAP. We proceeded with a high-resolution computed tomography (HRCT) scan to evaluate the extension of the mass and to look for bone involvement prior to the removal of the tumor.

**Figure 1:**
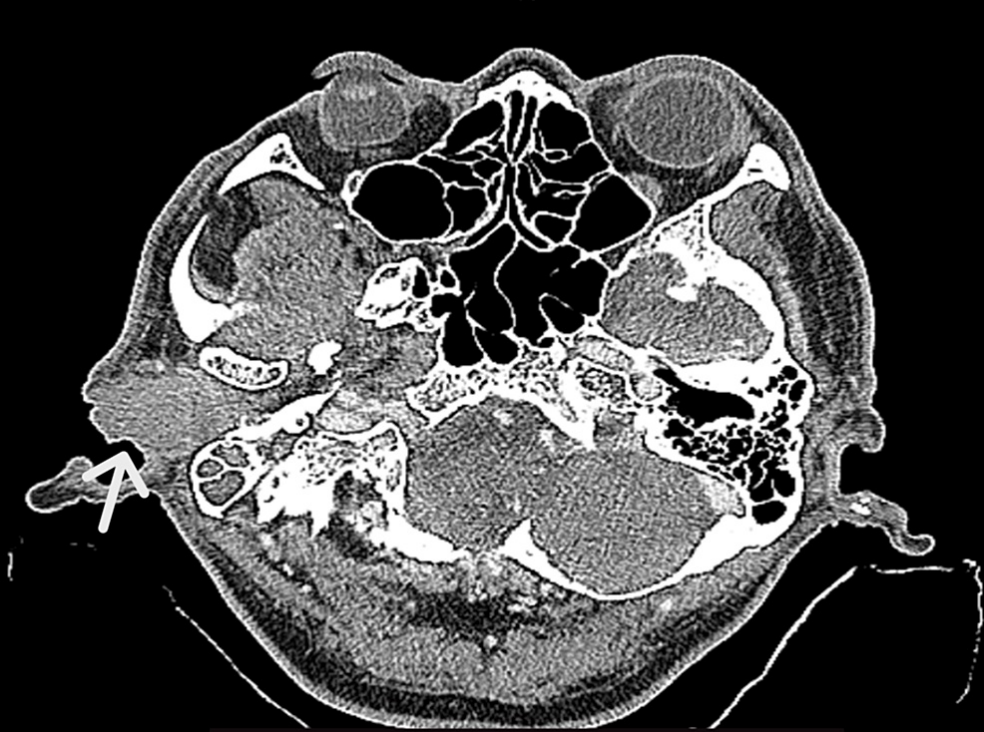
Axial view (bone window) a mildly enhancing mass with no bony wall erosions

HRCT temporal revealed a mildly enhancing soft tissue mass at the right EAC with involvement of the bony and cartilaginous part. It measured about 2.5 x 2.9cm at the cartilaginous part. It was anteriorly related to the mandibular condyle and proximal Eustachian tube and posteriorly abutting the anterior wall of the mastoid air cell. There were no bony erosions of the ear canal and the anterior wall of the mastoid air cell (Figure 1).

Examination under anesthesia and excision of mass were performed through an end-aural approach. Intraoperatively, the mass was occupying the entire EAC and was attached to the anterosuperior wall of the EAC, the posterior wall was not involved. The tumor was removed; post-removal, the right tympanic membrane was intact and the walls of the EAC were not eroded.

**Figure 2:**
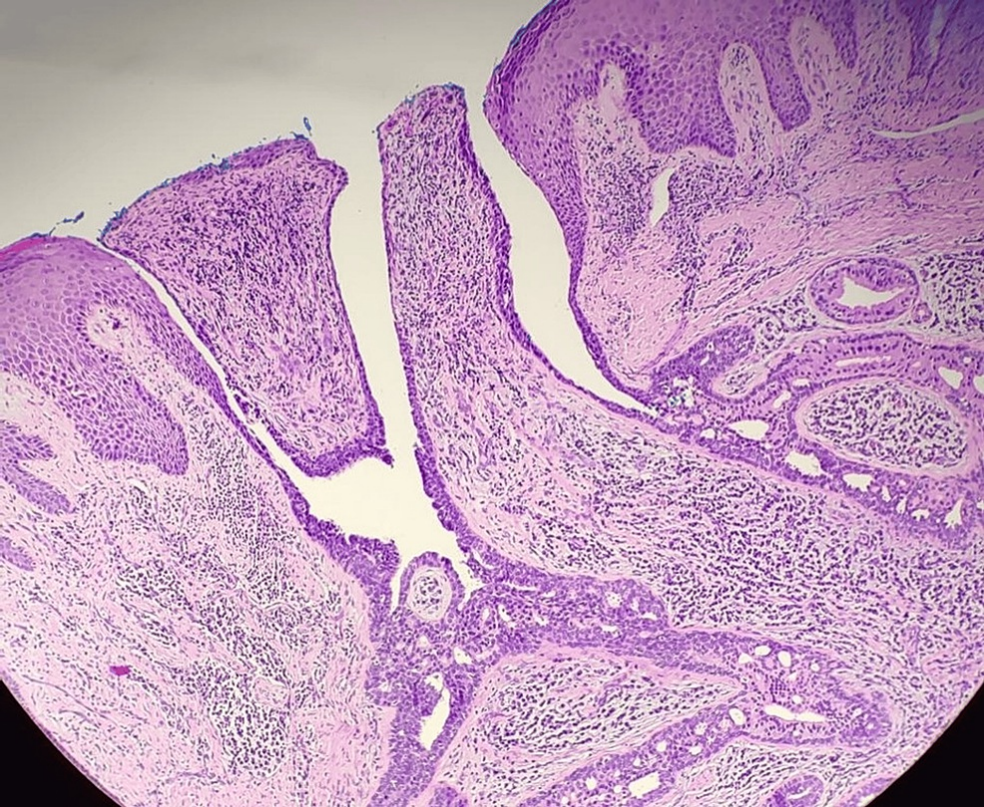
The lesion is connected focally to epidermis and composed of two layers of cells forming dilated tubular and papillary structures (H&E, 40x magnification). H&E - hematoxylin and eosin staining

**Figure 3:**
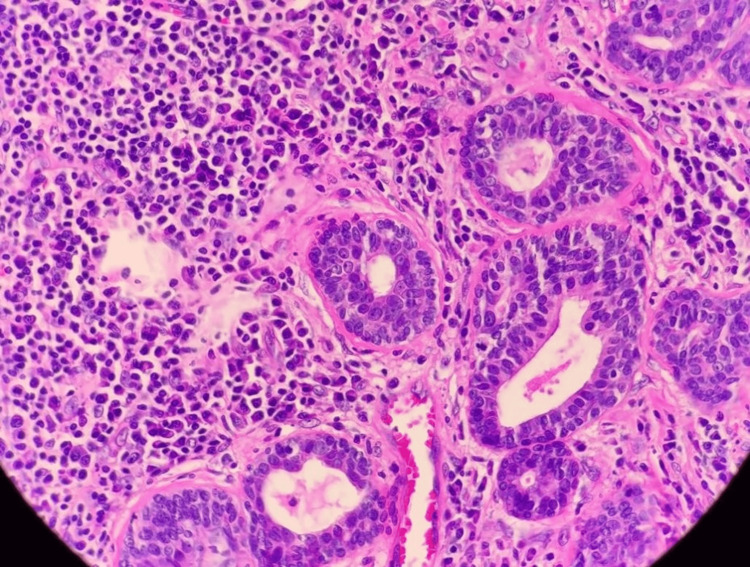
The luminal cells are columnar and the outer layer consists of myoepithelial cells. The stroma contains numerous plasma cells (H&E, 100x magnification). H&E - hematoxylin and eosin staining

Microscopic examinations revealed a skin-covered tissue enveloped by hyperkeratotic and acanthotic epidermis associated with an endophytic crateriform lesion, connected focally to the epidermis. The lesion is composed of dermal arborizing proliferation forming tubular and papillary structures, some of which appear cystically dilated. The tubules and papillary structures are lined by two-tiered luminal cuboidal to columnar cells with outer flattened to cuboidal myoepithelial cells. In areas, the luminal epithelium exhibits apocrine differentiation as characterized by ample eosinophilic cytoplasm with central, round nuclei and prominent nucleoli. The surrounding stroma shows moderate to dense lymphoplasma cell infiltration, especially at the periglandular region with scattered lymphoid follicles formation. No mitotic activity, cytological atypia, or evidence of malignancy is seen (Figures 2, 3). The resection lines were free of tumor tissue.

Postoperatively, there was an improvement in the hearing of the right ear; the audiometry showed mild sensorineural hearing loss similar to the hearing level of the left ear. After 10 months of monitoring, the patient had no signs of tumor recurrence.

## Discussion

SCAP is a rare hamartomatous adnexal tumor with apocrine and eccrine differentiation. Stokes and Werther described it in 1917 as “nevus syringadenomatosus papilliferum” because it was caused by a preexisting nevus sebaceous in nearly one-third of the cases. SCAP is a frequent sweat gland tumor of the face and scalp, whereas EAC and pinna involvement is uncommon. This tumor rarely appears on the eyelids, trunk, genitalia, or lower extremities [8]; it usually develops on the head and neck. The solitary nodular form exhibits a preference for the trunk [8], the plaque type which manifests as a hairless area on the scalp, is frequently associated with a sebaceous nevus of Jadassohn [9], and lesions on the face and neck region are typical of the linear type. A presentation with multiple lesions is rare, and even more uncommon if those lesions arise outside the head and neck region [9]. The nodular type of lesion as mentioned shows predilection in the trunk but in our case, it was present in the EAC.

SCAP is a benign ceruminous gland neoplasm with extensive papillary growth of epithelial elements down into the dermis [1]. Ceruminous glands are generally found in the outer one-third to one-half of the skin of the EAC, at the cartilaginous region. It is often assumed that such glands do not exist in the bony canal. However, multiple case reports have established that SCAP can also involve or arise from the osseous portion of the EAC [10], which is also demonstrated by the SCAP in our case report. Other regions of the ear, particularly the middle ear and pinna, have also been affected [10].

SCAP is primarily a pediatric tumor, manifesting either at birth or at puberty. It is present at birth in around 50% of individuals affected, and the tumor develops before puberty in the remaining 15%-30% [9]. In other cases, they appear from birth or in early childhood but grow in size during puberty. However, like in our case, the majority of cases in the literature have been documented in adult males [5]. SCAP involving the EAC of elderly patients is rare [10].

This benign tumor is initially mostly asymptomatic. Hearing loss, ear discharge, and bleeding are some of the symptoms associated with this tumor. Patients also present with otalgia due to mass effect and auditory canal obstruction by the neoplasm. Because of the restricted anatomical space of the EAC, tumor size ranges from 0.4cm to 2.0 cm [6]. Our patient presented with a growing ear tumor that eventually obstructed the EAC, causing hearing loss. Mild to moderate hearing loss is the most consistent symptom of EAC SCAP [10]. The lesions have ranged in size from 0.8cm to 4 cm, with the largest being measured by magnetic resonance imaging (MRI) in a case report by Kamakura et al. [11].

SCAP is a histopathological diagnosis, and the significance of radiography is not established [5]. However, HRCT and MRI are important modalities that can be used to aid in the diagnosis of EAC tumors. HRCT of the temporal bone can help describe the dimensions of the lesion, show involvement of the middle ear as well, and rule out malignancy which would be indicated by bone erosions. The MRI characteristics of an EAC SCAP were reported by Kamakura et al.: intermediate signal intensities on T1- and T2-weighted images, and mild augmentation on gadolinium-enhanced T1-weighted images [11].

Due to the heterogeneity of clinical symptoms, neoplasms emerging from the EAC ceruminous glands pose a diagnostic difficulty [12]. SCAP needs to be differentiated from the malignant tumors of the ceruminous glands; ceruminous adenocarcinoma, ceruminous adenoid cystic carcinoma, and ceruminous mucoepidermoid carcinoma. It can also co-exist with other types of benign lesions of the EAC such as chronic granular otitis externa with myringitis, lipomatous apocrine adenoma, tubular apocrine adenoma, and middle ear cholesteatoma [10]. Benign differential diagnoses for SCAP also include other benign tumors of ceruminous glands; ceruminous adenoma and ceruminous pleomorphic adenoma, cylindroma, papilloma, verrucous nevus, basaloid follicular hamartoma, pyogenic granuloma, neuroendocrine adenoma of the middle ear, parotid pleomorphic adenoma, meningioma and paraganglioma [3]. SCAP may also be associated with malignant neoplasms; ductal cell carcinoma, basal cell carcinoma, verrucous carcinoma [9], squamous cell carcinoma, and SCAP, its malignant counterpart [5]. SCAP should also be distinguished from certain diseases, for example, tuberculosis [1].

Ceruminous gland lesions in general are distinguished by a dual cell population; an abluminal basal myoepithelial cell layer enclosing an inner layer of luminal secretory cells that carry ceroid pigment and exhibit apical caps with decapitation secretion [6]. Histological examination of hematoxylin and eosin-stained sections of SCAP reveals the presence of unique papillary projections lined by a cuboidal myoepithelial layer surrounding columnar luminal cells with occasional decapitation secretions [10], allowing for the diagnosis of ceruminous SCAP without the use of immunohistochemistry. Histopathology of our patient's tumor also reported two-tiered luminal cuboidal to columnar cells with outer cuboidal myoepithelial cells. These were accompanied by a dense plasmacytic infiltrate in the fibrovascular stroma [6], which is also evident in our case.

Ceruminous SCAP is a histological diagnosis; samples can be obtained by incisional or excisional biopsy. A preoperative incisional biopsy could be performed to establish diagnosis however the sample obtained might miss the intraglandular papillary structures and the presence of an intralesional cyst that will aid in diagnosis. As all ceruminous gland tumors including SCAP have the potential for malignant transformation [13] and are associated with other malignant neoplasms, incisional biopsies carry the risk of missing these diagnoses. Moreover, incisional biopsies could also cause hemorrhage or facial paralysis in glomus tumors or schwannoma of the facial nerve [7]. As SCAP contains both solid and cystic parts, complete removal of the tumor will give a conclusive diagnosis. Excisional biopsies are also recommended as recurrences and malignant transformation can occur in incompletely removed tumors [3]. Surgical excision is considered the mainstay of treatment, but carbon dioxide lasers have also been used to treat SCAP, especially in pediatric patients [5]. Radiation and chemotherapy are not required to treat these neoplasms [10]. Periodic monitoring is recommended following the removal of the tumor as recurrences can occur in patients with positive surgical margins. In our patient, an incisional biopsy was done to establish a preoperative diagnosis and was followed through by an excisional biopsy to remove the tumor with resection lines being free of tumor tissue. There was no evidence of recurrence in our patient after 10 months of monitoring.

## Conclusions

SCAP is a rare and benign tumor of the ceruminous gland that can arise from the cartilaginous and bony portion of the EAC. Excisional biopsy with histopathological analysis is the mainstay treatment for an accurate diagnosis due to its non-specific clinical presentations however radiological examinations could be done to assess the margins of tumors preoperatively. A complete excision of the tumor is required to avoid recurrences and malignant transformation in tumor removals with positive surgical margins. Clinical follow-ups are recommended in such cases.
